# HOI-02 induces apoptosis and G2-M arrest in esophageal cancer mediated by ROS

**DOI:** 10.1038/cddis.2015.227

**Published:** 2015-10-15

**Authors:** C Zhang, K Liu, K Yao, K Reddy, Y Zhang, Y Fu, G Yang, T A Zykova, S H Shin, H Li, J Ryu, Y-n Jiang, X Yin, W Ma, A M Bode, Z Dong, Z Dong

**Affiliations:** 1 Department of Cellular and Molecular Biology, The Hormel Institute, University of Minnesota, Austin, MN, USA; 2 Department of Pathology and Pathophysiology, Basic Medical College, Zhengzhou University, Zhengzhou, China; 3 Department of Molecular Pathology, The Affiliated Cancer Hospital, Zhengzhou University, Zhengzhou, China; 4 China-US (Henan) Hormel Cancer Institute, Zhengzhou, China; 5 Program in Biomedical Informatics and Computational Biology, University of Minnesota, Minneapolis, MN, USA

## Abstract

Reactive oxygen species (ROS) are chemically reactive molecules that perform essential functions in living organisms. Accumulating evidence suggests that many types of cancer cells exhibit elevated levels of ROS. Conversely, generation of ROS has become an effective method to kill cancer cells. (E)-3-hydroxy-3-(4-(4-nitrophenyl)-2-oxobut-3-en-1-yl) indolin-2-one, which is an NO_2_ group-containing compound designated herein as HOI-02, generated ROS and, in a dose-dependent manner, decreased esophageal cancer cell viability and inhibited anchorage-independent growth, followed by apoptosis and G2-M arrest. Moreover, results of an *in vivo* study using a patient-derived xenograft mouse model showed that HOI-02 treatment suppressed the growth of esophageal tumors, without affecting the body weight of mice. The expression of Ki-67 was significantly decreased with HOI-02 treatment. In addition, the phosphorylation of c-Jun, and expression of p21, cleaved caspase 3, and DCFH-DA were increased in the HOI-02-treated group compared with the untreated control group. In contrast, treatment of cells with (E)-3-(4-(4-aminophenyl)-2-oxobut-3-en-1-yl)-3-hydroxyindolin-2-one, which is an NH_2_ group-containing compound designated herein as HOI-11, had no effect. Overall, we identified HOI-02 as an effective NO_2_ group-containing compound that was an effective therapeutic or preventive agent against esophageal cancer cell growth.

Esophageal cancer remains one of the most lethal cancers worldwide with its incidence on the rise. It is the fourth most frequently diagnosed cancer and the fourth leading cause of cancer death in China.^1^ In 2014 alone, esophageal cancer affected over 18 000 people across the United States and approximately 15 500 succumbed to this disease.^2^ Despite clinical advances in the field of oncology, esophageal cancer remains one of the leading causes of cancer-associated mortality. The overall 5-year survival rate for all patients with esophageal cancer is <20%.^3^ Owing to its aggressive nature and poor response to chemotherapy, esophageal cancer remains a challenging disease to treat.^2^ Therefore, research to identify and develop more effective drugs to prevent or treat esophageal cancer is urgently needed.

Reactive oxygen species (ROS) production is a common characteristic of most non-surgical therapeutic approaches, including chemotherapy and radiotherapy, against various cancers because of the ability of ROS to trigger cancer cell death.^4^ More ROS-generating agents with different mechanisms of action are needed to fully understand their potential application in cancer treatment.^5^ Inducing ROS generation is considered a novel approach in cancer treatment^6, 7^ and the advantage of this strategy lies in its selectivity. Cancer cells are usually under oxidative stress and, hence, already contain a relatively high basal level of ROS.^8, 9^ A small induction of ROS in tumor cells may push the level of ROS over the threshold of life and death to induce cell death, whereas normal cells can better tolerate the oxidative insults because of their lower basal level of ROS and stronger antioxidant capacities.^4^ Hence, designing and producing drugs that can generate ROS to improve esophageal cancer treatment would be helpful and important.

In this study, we found that HOI-02, which was synthesized in our laboratory, could dose dependently induce ROS production corresponding with decreased esophageal cancer cell viability and inhibition of anchorage-independent cell growth. Biologic testing further confirmed that HOI-02 potently inhibited esophageal cancer cell growth by inducing apoptosis and G2-M arrest *in vitro* and *in vivo*. Signaling, involving c-Jun N-terminal kinases (JNKs), AP-1, caspase 3, ATM, p53 and p21, was activated in cells treated with HOI-02. Thus, HOI-02 may be useful in prevention or treatment of esophageal cancer.

## Results

### HOI-02 decreases esophageal cancer cell viability and inhibits anchorage-independent cell growth

**Figure 1:**
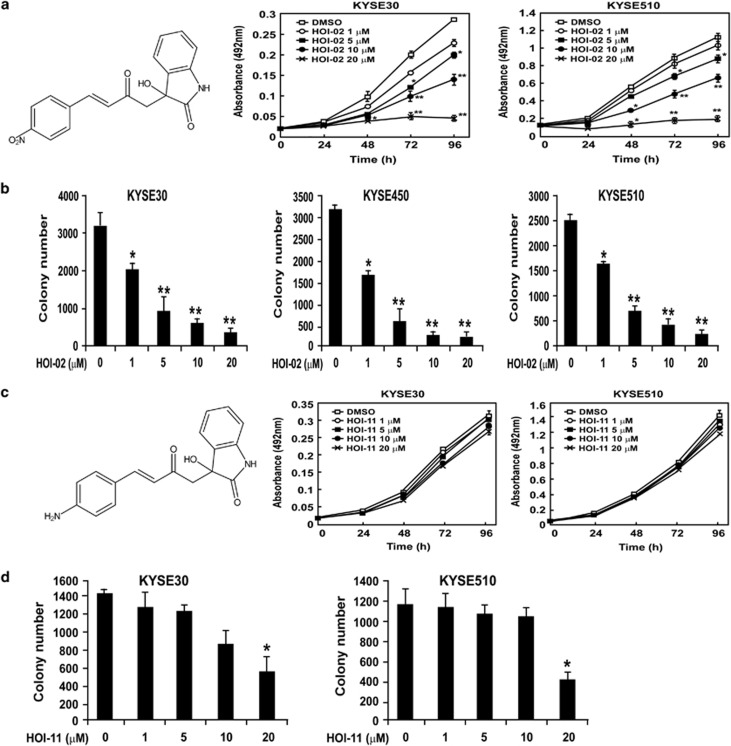
HOI-02 decreases viability of esophageal cancer cells and their anchorage-independent growth. (**a**) Chemical structure of HOI-02 (left panel). HOI-02 decreases KYSE30 (middle panel) and KYSE510 (right panel) esophageal cancer cell viability in a dose-dependent manner. KYSE30 and KYSE510 cells (1 × 10^3^ per well) were treated with the indicated doses of HOI-02 for the specified times. The absorbance was measured as described in Materials and methods section. Data are shown as mean values±S.D. and the asterisks (*, **) indicate a significant (*P*<0.05, *P*<0.01, respectively) decrease in viability of cells treated with HOI-02 compared with untreated control cells. (**b**) HOI-02 suppresses anchorage-independent growth of esophageal cancer cells. Colony numbers are shown as mean values±S.D. from three independent experiments. The asterisks (*, **) indicate a significant (*P*<0.05, *P*<0.01, respectively) decrease in colony numbers in cells treated with HOI-02 compared with the DMSO-treated group. (**c**) Chemical structure of HOI-11 (left panel). HOI-11 has no effect on viability of KYSE30 (middle panel) or KYSE510 (right panel) esophageal cancer cells. KYSE30 or KYSE510 cells (1 × 10^3^ per well) were treated with the indicated doses of HOI-11 for the specified times. The absorbance was measured as described in Materials and methods section. Data are shown as mean values±S.D. (**d**) Only the highest dose of HOI-11 suppresses anchorage-independent growth of esophageal cancer cells. Colony numbers are shown as mean values±S.D. from three independent experiments. The asterisk (*) indicates a significant (*P*<0.05) decrease in colony numbers of cells treated with HOI-11 compared with the DMSO-treated group

HOI-02 (Figure 1a, left panel, Supplementary Figure S1A) is a novel compound synthesized in our laboratory. Our data indicated that the viability KYSE30 (Figure 1a, middle panel) and KYSE510 (Figure 1a, right panel) esophageal cancer cells was decreased by HOI-02 in a dose-dependent manner. Furthermore, we examined the effect of HOI-02 on anchorage-independent growth of three esophageal cancer cell lines. We found that HOI-02 inhibited anchorage-independent growth, leading to a dose-dependent reduction of colony formation (Figure 1b, Supplementary Figure S1B). At the same time, we examined the effect of another compound, HOI-11 (Figure 1c, left panel, Supplementary Figure S2A), which contains an NH_2_ group instead of the NO_2_ group. In contrast to HOI-02, HOI-11 had little or no effect on viability (Figure 1c, middle and right panels) or anchorage-independent growth (Figure 1d, Supplementary Figure S2B) of esophageal cancer cells. Only the highest concentration (20 *μ*M) could inhibit colony formation (Figure 1d).

### HOI-02 induces apoptosis and G2-M arrest in esophageal cancer cells

**Figure 2:**
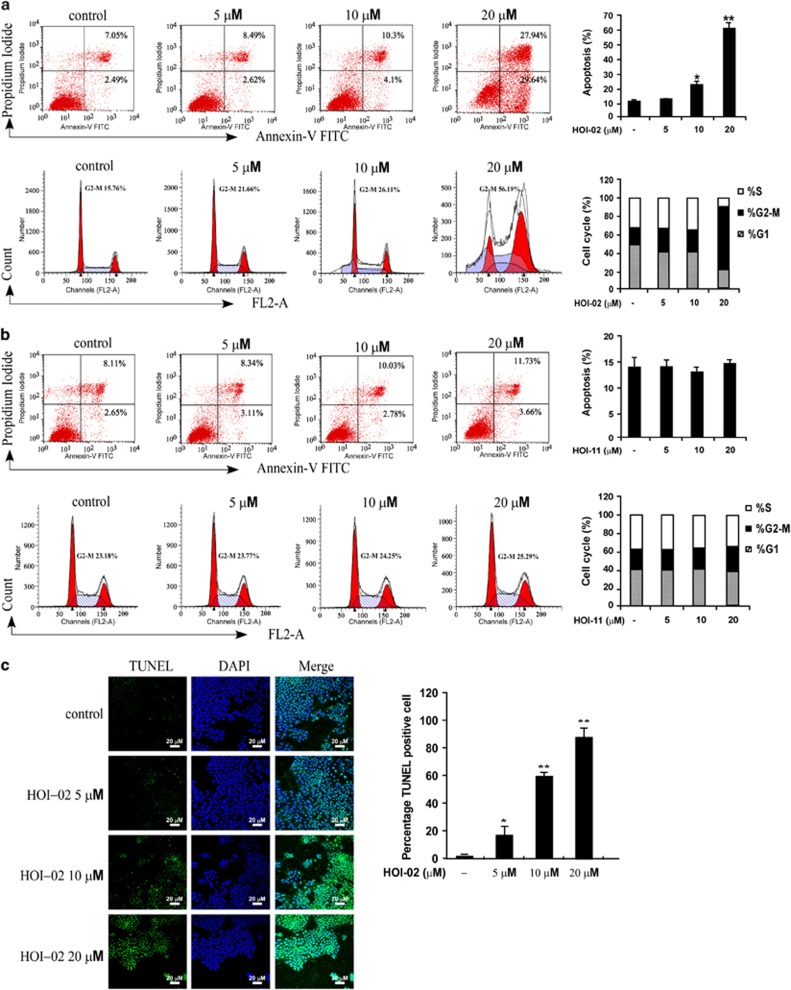
HOI-02 induces apoptosis and G2-M arrest in esophageal cancer cells. (**a**) Flow cytometry analysis of apoptosis (upper panels) and cell cycle (lower panels) in KYSE510 cells with treatment of HOI-02. Percent apoptosis is shown as mean values±S.D. from three independent experiments. The asterisks (*, **) indicate a significant (*P*<0.05, *P*<0.01, respectively) difference in apoptosis in cells treated with HOI-02 compared with the DMSO-treated group. (**b**) Flow cytometry analysis of apoptosis (upper panels) and cell cycle (lower panels) in KYSE510 cells treated with HOI-11. Percent apoptosis is shown as mean values±S.D. from three independent experiments. (**c**) Immunocytochemical staining of TUNEL-positive cells treated with HOI-02. DAPI was used for counter-staining cells. The experiment was repeated three times and representative results are shown. The asterisks (*, **) indicate a significant (*P*<0.05, *P*<0.01, respectively) increase in TUNEL-positive cells

To study the mechanism of the inhibitory effects of HOI-02, apoptosis and cell cycle distribution were measured by flow cytometry using annexin V/propidium iodide (PI) staining. Treatment of KYSE510 cells with HOI-02 (0, 5, 10 or 20 *μ*M) for 24 h resulted in increased numbers of apoptotic cells ranging from 9.54 to 57.58% (Figure 2a, upper panels) and amplification of G2-M phase arrest from 15.76 to 56.19% (Figure 2a, lower panels). These results confirm that HOI-02 induces apoptosis and G2-M phase arrest in esophageal cancer cells and thus inhibits their growth. We also treated cells with HOI-11 under the same conditions as HOI-02 and found that HOI-11 could not induce apoptosis or G2-M arrest (Figure 2b). Furthermore, we quantified the level of apoptosis using the TUNEL assay (terminal deoxynucleotidyl transferase dUTP nick-end labeling) and found that TUNEL-positive cancer cells were significantly increased dose dependently with HOI-02 treatment compared with the untreated control group (Figure 2c). This result confirms that HOI-02 induces apoptosis of esophageal cancer cells.

### ROS are involved in HOI-02-induced apoptosis and G2-M arrest in esophageal cancer cells

**Figure 3:**
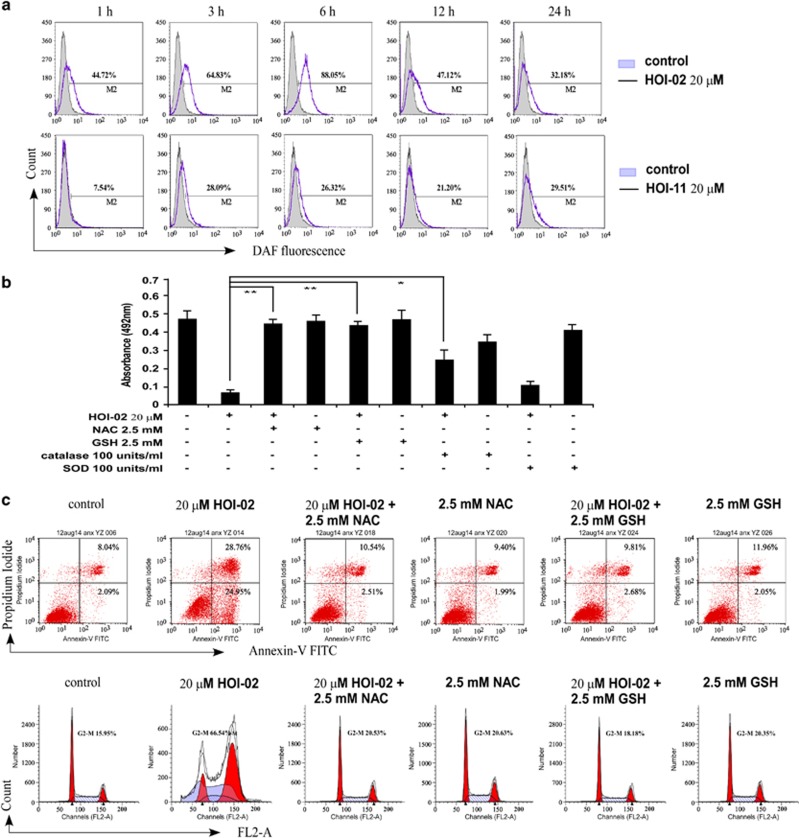
ROS are involved in HOI-02-induced apoptosis and G2-M arrest of esophageal cancer cells. (**a**) KYSE510 cells were treated with 20 *μ*M HOI-02 for the indicated times and the production of intracellular ROS was detected by flow cytometry using DCFH-DA. (**b**) HOI-02 decreases viability of KYSE510 esophageal cancer cells even in the presence of SOD, but not with NAC or GSH. KYSE510 cells (1 × 10^3^ per well) were treated as indicated for 24 h. The absorbance was measured as described in Materials and methods section. Data are shown as mean values±S.D. and the asterisks (*, **) indicate a significant (*P*<0.05, *P*<0.01, respectively) decrease in viability of treated cells compared with the untreated control cells. (**c**) KYSE510 esophageal cancer cells (5 × 10^5^) were treated with 20 *μ*M HOI-02 for 24 h with or without 2.5 mM NAC or GSH. Apoptosis (upper panels) and cell cycle (lower panels) were analyzed by flow cytometry

Treatment of KYSE510 cells with 20 *μ*M HOI-02 increased the generation of ROS as early as 1 h (44.72%) and the level of ROS peaked at 6 h (88.05% Figure 3a, upper panels). In contrast, treatment with 20 *μ*M HOI-11  had a smaller effect (7.54% at 1 h and 26.32% at 6 h, Figure 3a, lower panels). Notably, treatment of KYSE510 cells with free radical scavengers, NAC, glutathione (GSH) or catalase, prevented the decrease in viability (Figure 3b), apoptosis (Figure 3c upper panels) and G2-M arrest (Figure 3c, lower panels) induced by HOI-02. These results suggest that ROS have a role in the action of HOI-02 against esophageal cancer cell growth.

### EPR analysis confirms the involvement of ROS in the anticancer activity of HOI-02

**Figure 4:**
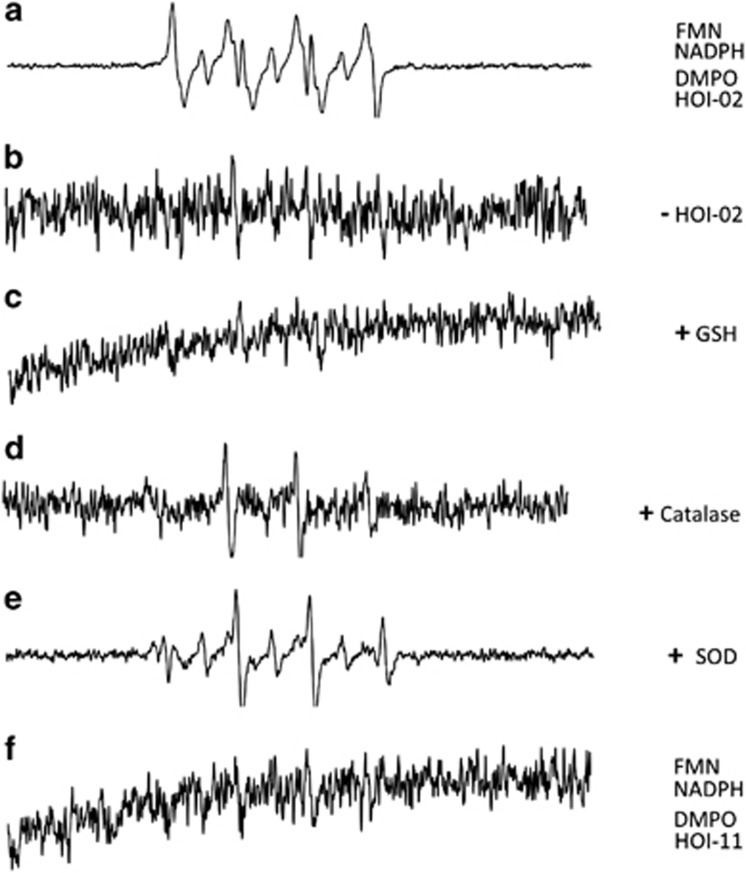
EPR signals generated by DMPO spin adducts in the HOI-02/NADPH/FMN system. EPR spectra were obtained from incubation mixtures containing phosphate buffer (pH 7.4, 50 *μ*M diethylenetriamine pentaacetate (DTPA)) and 100 mM DMPO: (**a**) HOI-02 (20 *μ*M), DMPO (100 mM), NADPH (0.4 mM) and FMN (7 *μ*M). (**b**) Same as **a**, but without compound HOI-02. (**c**) Same as **a**, but with 2.5 mM GSH. (**d**) Same as **a**, but with 100 units/ml of catalase. (**e**) Same as **a**, but with 100 units/ml of SOD. (**f**) Same as **a**, but compound HOI-02 is replaced with HOI-11

To further confirm that ROS are involved in HOI-02-induced apoptosis and G2-M arrest in esophageal cancer cells, the electron paramagnetic resonance (EPR) spin trapping regent, 5,5-dimethyl-1-pyrroline-*N*-oxide (DMPO), was used to detect ROS generation in the presence of HOI-02. In this experimental system, DMPO was added following the addition of nicotinamide adenine dinucleotide phosphate (NADPH) to phosphate-buffered saline (PBS) containing FMN and HOI-02. The reaction was incubated in an anaerobic environment for 15 min to determine whether these aromatic nitro-containing compounds would generate their corresponding nitro anion free radical.^10^ The reaction containing riboflavin-5′-monophosphate (FMN), NADPH, DMPO and HOI-02 generated a strong radical signal in this system (Figure 4a). In contrast, when HOI-02 was removed, a free radical signal was not detectable (Figure 4b). GSH or catalase, but not superoxide dismutase (SOD), could significantly decrease the signal, suggesting that that H_2_O_2_ or glutathione, but not O_2_–, may have a critical role in the effects of HOI-02 treatment (Figures 4c–e). The generation of other free radicals such as NO and NO_2_ was not detected with EPR and iNOS was also not induced with HOI-02 treatment as indicated by western blot analysis (Supplementary Figure S3). In addition, no radical signal was detected when HOI-02 was replaced with HOI-11 (Figure 4f). These studies indicate that ROS have a substantial role in HOI-02 treatment.

### HOI-02-induced apoptosis is mediated through the induction of AP-1 activity and the modulation of caspase activity

**Figure 5:**
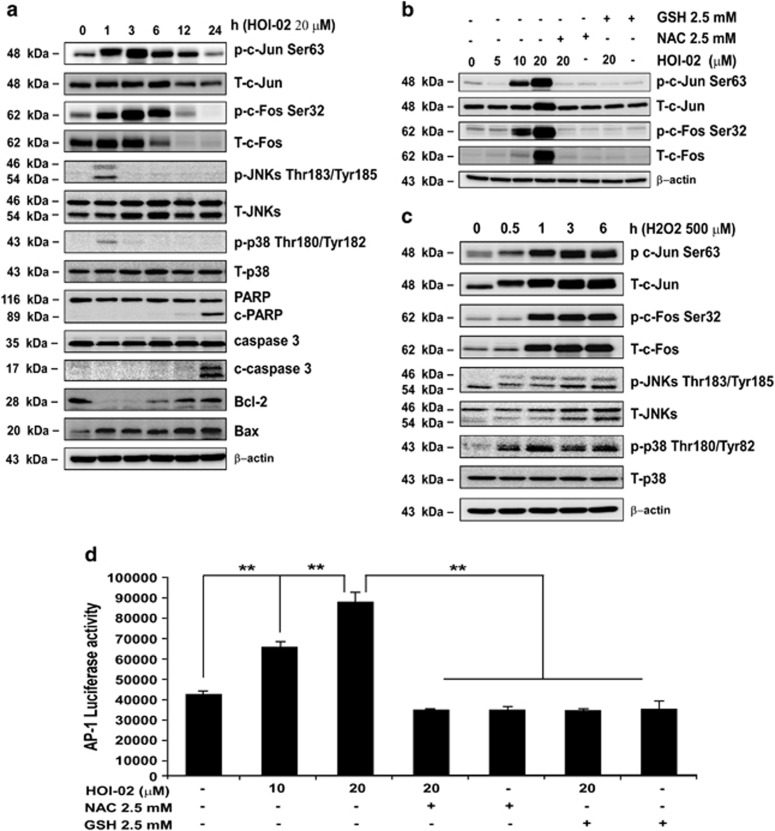
HOI-02-induced apoptosis is mediated through induction of AP-1 activity and the modulation of caspase expression. (**a**) KYSE510 cells (1 × 10^6^) were treated with 20 *μ*M HOI-02 for the indicated times. The expression of apoptosis-related proteins was determined by western blot analysis using specific antibodies. *β*-Actin was detected in the same membrane and served as a loading control. Data shown are representative of results from triplicate independent experiments. (**b**) KYSE510 cells (1 × 10^6^) were treated with various doses of HOI-02 (0, 5, 10 or 20 *μ*M), 2.5 mM NAC or 2.5 mM GSH for 24 h. The expression of proteins was determined by western blot analysis using specific antibodies. (**c**) ROS induce phosphorylation of c-Jun and c-Fos in esophageal cancer cells. After treatment with 500 *μ*M H_2_O_2_ for the indicated times, the expression of proteins was determined by western blot analysis using specific antibodies. (**d**) HOI-02 induces AP-1 luciferase activity and the effect is blocked by NAC or GSH in KYSE510 esophageal cancer cells. KYSE510 cells were transiently transfected with the *AP-1 luciferase reporter* gene construct and incubated with HOI-02 (0, 10 or 20 *μ*M), 2.5 mM NAC or 2.5 mM GSH. Luciferase activity was measured as described in Materials and methods section

To delineate the mechanism of HOI-02-induced apoptosis, the expression of apoptosis-associated proteins was evaluated in KYSE510 cells after exposure to 20 *μ*M HOI-02 for various times (0, 1, 3, 6, 12 or 24 h). Results indicate that HOI-02 treatment increased the expression of cleaved caspase 3, cleaved poly ADP-ribose polymerase (PARP) and Bax. These changes were accompanied by an increased expression of phosphorylation of c-Jun (Ser63), c-Fos (Ser32), JNKs (Thr183/Tyr185) and p38 (Thr180/Tyr182) (Figure 5a). Moreover, we also observed that phosphorylation of c-Jun (Ser63) and c-Fos (Ser32) disappeared when treated with HOI-02 and NAC or GSH simultaneously (Figure 5b). Furthermore, in cells treated with H_2_O_2_ (500 *μ*M), phosphorylation of c-Jun (Ser63), c-Fos (Ser32), JNKs (Thr183/Tyr185) and p38 (Thr180/Tyr182) was also increased over time (Figure 5c). c-Jun and c-Fos commonly interact to form the heterodimeric transcription factor, activator protein-1 (AP-1) and ROS have been reported to mediate AP-1 activity.^11^ Herein, we showed that HOI-02 could significantly induce AP-1 luciferase activity dose dependently and NAC or GSH could block the effect (Figure 5d). These results further suggest that HOI-02 exerts its effects by generating ROS, which then contribute to the activation of key apoptosis-related proteins.

### HOI-02-induced G2-M arrest is mediated through p21-related signaling

**Figure 6:**
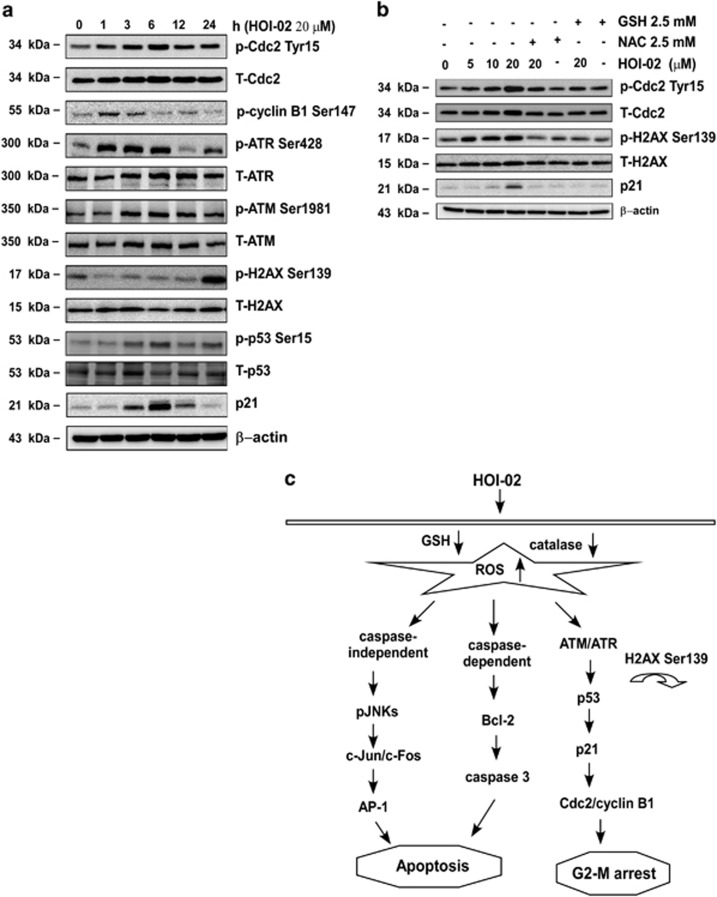
HOI-02-induced G2-M arrest is mediated through a p21-related pathway. (**a**) KYSE510 cells (1 × 10^6^) were treated with 20 *μ*M HOI-02 for the indicated times. The expression of cell cycle-related proteins was determined by western blot analysis using specific antibodies. *β*-Actin was detected in the same membrane and served as a loading control. Data shown are representative of results from triplicate independent experiments. (**b**) KYSE510 cells (1 × 10^6^) were treated with various doses of HOI-02 (0, 5, 10 or 20 *μ*M), 2.5 mM NAC or 2.5 mM GSH for 24 h. The expression of proteins was determined by western blot analysis using specific antibodies. (**c**) A proposed model of the mechanisms of apoptosis and G2-M phase arrest induced by HOI-02 in esophageal cancer cells

We then examined the effect of 20 *μ*M HOI-02 at various time points on the expression of several proteins known to be associated with G2-M phase in KYSE510 cells. We found that phosphorylation of Cdc2 (Tyr15), ATR (Ser428), ATM (Ser1981), H2AX (Ser139), p53 (Ser15) and total p21 were increased, whereas phosphorylation of cyclin B1 (Ser147) was decreased with HOI-02 treatment (Figure 6a). These changes in protein expression could result in G2-M arrest. We also examined the effect of HOI-02 treatment with and without NAC or GSH and found that the increases in phosphorylation of Cdc2 (Tyr15), H2AX (Ser139) and total p21 were attenuated when GSH or NAC was included in the reaction (Figure 6b). Overall, our results in esophageal cancer cells treated with HOI-02 suggest that ROS activate AP-1 and cleaved caspase 3, which contribute to apoptosis, and also p21 signaling, which participates in G2-M arrest (Figure 6c).

### HOI-02 suppresses tumor growth *in vivo* by generating ROS and activating AP-1, caspase 3 and p21 signaling

**Figure 7:**
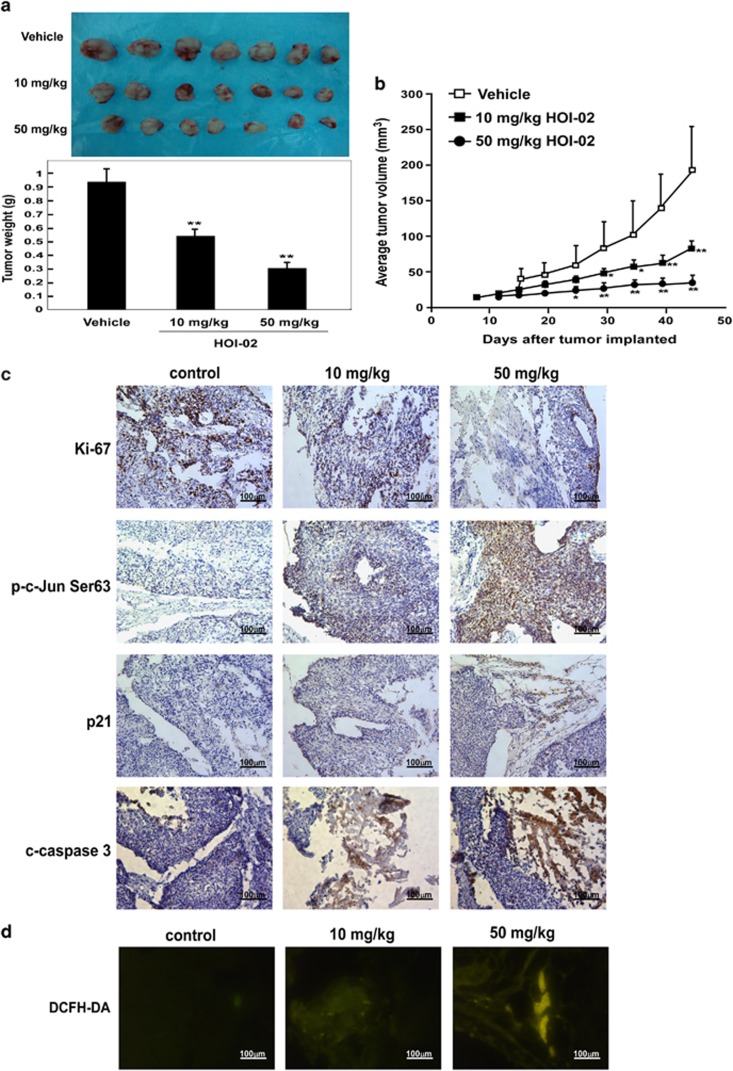
HOI-02 suppresses tumor growth *in vivo* by generation of ROS and activation of AP-1, cleaved caspase 3 and p21. (**a**) The total average tumor weight in the HOI-02-treated group is significantly less than that of the vehicle-treated group. Tumors were extracted and weighed after mice were killed. Data are shown as mean values±S.D. The asterisks (**) indicate a significant decrease in tumor weight (*P*<0.01) in the HOI-02-treated group compared with the vehicle-treated group. (**b**) HOI-02 treatment suppresses tumor volume compared with the vehicle-treated group. Tumor volume was measured and recorded as described in Materials and methods section. The asterisks (*, **) indicate a significant decrease in tumor size (*P*<0.05, *P*<0.01, respectively) in the HOI-02-treated group compared with the vehicle group. (**c**) Immunohistochemical analysis of tumor tissues. Treated or untreated groups of mice were euthanized and tumors extracted. Esophageal cancer tissue slides were prepared with paraffin sections after fixation with formalin and then stained to detect the indicated proteins. (**d**) Fluorescent microscopic observation of sections stained with DCFH-DA, which reflects the level of ROS in the tissues

We evaluated the effect of HOI-02 on growth of esophageal cancer patient-derived xenograft (PDX) growth. Treatment of mice with HOI-02 reduced tumor weight dose dependently compared with the untreated control (Figure 7a; *P*<0.01) and the mean tumor volume in the vehicle-treated group increased faster than that in the HOI-02-treated group (Figure 7b; *P*<0.01). Moreover, immunohistochemical analysis of tumors showed that the expression of Ki-67, a cell proliferation marker, was markedly decreased and phosphorylation of c-Jun (Ser63) and expression of total p21 and cleaved caspase 3 were increased in tumors treated with HOI-02 compared with the untreated controls (Figure 7c). In addition, immunofluorescence results show that DCFH-DA was significantly increased in HOI-02-treated tumors compared with the controls (Figure 7d), which indicates the presence of ROS. Collectively, these findings provide evidence supporting the idea that the anticancer efficacy of HOI-02 is mediated *in vivo* by generation of ROS resulting in increased cleavage of caspase 3, induction of AP-1 and enhanced p21 signaling, all of which contribute to the inhibition of esophageal cancer cell growth.

## Discussion

Accumulating evidence suggests that many types of cancer cells exhibit increased levels of ROS.^9^ ROS, like hydrogen peroxide (H_2_O_2_) and others, can act as second messengers in cellular signaling.^12, 13^ ROS regulate protein activity through reversible oxidation of proteins such as tyrosine phosphatases, tyrosine kinases, transcription factors and receptor tyrosine kinases.^14, 15^ In this study, we found that HOI-02, which contains an NO_2_ group, could increase the generation of ROS in esophageal cancer cells. The nitro group in HOI-02 can undergo enzymatic one-electron reduction to form a nitro radical anion and one-electron reduction has been described in microsomes and mitochondria and with purified enzymes.^16^ Under aerobic conditions, molecular oxygen oxidizes the nitro anion radical, resulting in a redox cycle with regeneration of the nitro compound and production of superoxide anion, whose dismutation yields H_2_O_2_ (Ask *et al.*^17^) (Supplementary Figure S2C). Our data indicated that H_2_O_2_ is the most likely ROS species generated by HOI-02 against esophageal cancer cell growth. As shown in the Figures 3b and c, GSH, but not SOD, could significantly inhibit cell viability, apoptosis and G2-M arrest. Also, as shown in Figure 4, we found that GSH or catalase, but not SOD, could significantly decrease the radical signal. The sensitivity to catalase indicates that the radical signal is likely derived from H_2_O_2_. In addition, the generation of other free radicals such as NO and NO_2_ was not detected with EPR and iNOS was also not induced with HOI-02 treatment as indicated by western blot analysis. These studies indicate that ROS have a significant role of HOI-02 treatment in esophageal cancer cells.

Induction of apoptosis has been a key strategy for cancer therapy. Successful apoptosis-inducing anticancer drugs cause tumor cells to die by either directly turning on pro-apoptotic pathways or turning off anti-apoptotic pathways.^18^ ROS have an important role in inducing apoptosis under both physiological and pathological conditions.^19, 20, 21, 22^ ROS are known to cause apoptosis both through caspase-dependent and caspase-independent cell signaling cascades.^23, 24^ Apoptotic cell death has traditionally been associated with caspase activation. The released apoptotic proteins initiate caspase activation and trigger caspase-mediated apoptotic DNA fragmentation and eventually cell death. In this study, we showed that HO1-02 induced caspase 3 and PARP cleavage (Figure 5a). JNKs are one of the major signaling molecules that are activated through the caspase-independent pathway.^25^ After activation by ROS, JNKs can stimulate AP-1, a transcription factor consisting of dimers of the Fos and Jun families of proteins. The activation of AP-1 appears to be at least partially responsible for apoptosis stimulated by HOI-02 (Figure 5d). AP-1 luciferase activity or phosphorylation of Fos and Jun induced by HOI-02 was attenuated by GSH or NAC, suggesting that HOI-02 induces esophageal cancer cell apoptosis through the generation of ROS.

Cancer progression is believed to involve the loss of checkpoint controls that regulate normal passage through the cell cycle. By supplementing existing cell cycle machinery with extrinsic cell cycle regulators, blocking initiation or progression of cancer may be possible. HOI-02 treatment caused G2-M cell cycle arrest, which could be beneficial in suppressing the progression of esophageal cancer (Figure 2a, lower panels). We observed that HOI-02 activated the ATM/p53-p21 signaling network (Figure 6a), which has been implicated in G2-M cell arrest.^26^ Generally, the activation of ATR is considered as a consequence of the formation of single-stranded DNA.^27^ The generation of single-stranded DNA in re-replicated cells may account for the induction of the G2-M checkpoint mediated by ATR. DNA re-replication continues as the cells are arrested in G2-M phase.^28^ In contrast, activation of ataxia telangiectasia mutated (ATM) is observed mostly in cells with double-stranded DNA breaks^27^ and is one of the proteins activated by ROS that is involved in cell cycle regulation.^14^ The mitogen-activated protein kinases (MAPKs), p38 and JNKs, have been implicated in apoptotic signaling in response to increased generation of ROS.^29^ Histone H_2_AX phosphorylation on Ser139 in response to DNA damage involves formation of DNA double-strand breaks.^30, 31^ This phosphorylation is mediated by ATM and ATR and was also triggered by HOI-02 and disappeared when cells were treated with GSH or NAC in combination with HOI-02 (Figure 6b).

For many years, mouse xenograft models implanted with human cancer cells lines have been used extensively for predicting responsiveness to anticancer target agents. In contrast, PDXs are based on the transfer of primary tumors directly from the human patient into an immune-deficient mouse. PDX models may be superior to traditional cell line xenograft models of cancer because they maintain more similarities to the parental tumors.^32^ PDX models also provide an invaluable assessment of tumor evolution and adaptive response to therapy.^33^ In this study, HOI-02 consistently and significantly inhibited growth of patient-derived esophageal cancers in nude mice (Figures 7a and b). Tissue samples from tumors treated with HOI-02 showed more intense staining with DCFH-DA compared with the untreated control (Figure 7d). Taken together, our cell-based studies and *in vivo* studies support the concept that HOI-02 treatment could effectively inhibit esophageal cancer cell growth by inducing apoptosis and cell cycle arrest.

## Materials and Methods

### Reagents and antibodies

RPMI-1640 medium and fetal bovine serum (FBS) were from Mediatech, Inc. (Manassas, VA, USA), *N*-acetyl cysteine (NAC), catalase, SOD and HiQ Standard Agarose were purchased from Sigma-Aldrich (St. Louis, MO, USA). Glutathione (GSH), DMPO, 2,7-dichlorodihydrofluorescein diacetate (DCFH-DA) and NADPH were obtained from Cayman Chemical (Ann Arbor, MI, USA). FMN was from TCI AMERICA (Portland, OR USA). Antibodies to detect total c-Jun, phosphorylated c-Jun (Ser63), total c-Fos, phosphorylated c-Fos (Ser32), total JNKs, phosphorylated JNKs (Thr183/Tyr185), total p38, phosphorylated p38 (Thr180/Tyr182), total ATR, phosphorylated ATR (Ser428), phosphorylated ATM (Ser1981), total H2AX, phosphorylated H2AX (Ser139), total p53, phosphorylated p53 (Ser15), total CDC2, phosphorylated CDC2 (Tyr15), phosphorylated cyclin B1 (Ser147), caspase 3 (Asp175), PARP, cleaved PARP (Asp214) and iNOS were purchased from Cell Signaling Technology, Inc. (Danvers, MA, USA). The antibodies to detect total ATM, Bax and p21 were obtained from Santa Cruz Biotechnology, Inc. (Santa Cruz, CA, USA).

### Cell culture

The KYSE510, KYSE30 and KYSE450 human esophageal cancer cell lines were cultivated in RPMI-1640 media supplemented with 10% heat-inactivated FBS. All cells used in these studies were maintained with antibiotics at 37 °C in a 5% CO_2_ humidified incubator according to American Type Culture Collection protocols (ATCC, Manassas, VA, USA). Cells were crytogenetically tested and authenticated before being frozen. Each vial of frozen cells was thawed and maintained for a maximum of 20 passages.

### MTS assay

KYSE510 or KYSE30 cells (1 × 10^3^) were seeded into 96-well plates in 100 *μ*l of 10% FBS/RPMI-1640 medium and incubated at 37 °C in a 5% CO_2_ humidified incubator. After culturing for 12 h, different concentrations of HOI-02 or HOI-11 and NAC, GSH, catalase, or SOD were added to each well. After incubation for another 24, 48, 72 or 96 h, 20 *μ*l of the CellTiter 96 Aqueous One Solution (Promega, Madison, WI, USA) were added to each well and cells were then incubated for an additional 1 h at 37 °C. Absorbance was measured at an optical density of 490 nm using the Thermo Multiskan plate-reader (Thermo Fisher Scientific, Waltham, MA, USA).

### Western blot analysis

Cellular proteins were extracted using cell lysis buffer (150 mM NaCl, 0.25% sodium deoxycholate, 50 mM Tris-HCl pH 8.0, 1% Nonidet P-40, 0.1% sodium dodecyl sulfate (SDS), 1 mM EDTA and protease inhibitor mixture). Protein concentration was measured using a protein assay kit (Bio-Rad, Hercules, CA, USA) and proteins were subjected to 10% SDS/polyacrylamide gel electrophoresis (PAGE). Proteins were then transferred onto polyvinylidene difluoride (PVDF) membranes (Amersham Biosciences, Piscataway, NJ, USA) and hybridized with the appropriate specific primary antibody and a horseradish peroxidase-conjugated secondary antibody. Antibody binding was conducted at 4 °C overnight and proteins were visualized using an enhanced chemiluminescence reagent and the ImageQuant LAS4000 system (GE Healthcare, Piscataway, NJ, USA).

### Flow cytometry analysis

KYSE510 cells were plated in 10-cm dishes and treated with vehicle (DMSO only), HOI-02 or HOI-11 for 24 h. Cells were then harvested and washed with PBS and subsequently stained with Annexin V-FITC and propidium iodide (MBL International Corp., Woburn, MA, USA). Cell cycle distribution or apoptosis was determined using the FACSCalibur flow cytometer (BD Biosciences, San Jose, CA, USA).

### Measurement of intracellular ROS by flow cytometry

After treatment for different times with HOI-02 or HOI-11, DCFH-DA was added to cells at a final concentration of 10 *μ*M. Cells were incubated at 37 °C in a 5% CO_2_ humidified incubator for 30 min. Cells were then washed with PBS, collected by centrifugation and analyzed on the FACSCalibur flow cytometer (BD Biosciences).

### EPR measurements

All EPR measurements were conducted using a Bruker EleXsys E500 spectrometer (Billerica, MA, USA) equipped with an ER 4122 SHQ spherical resonator (Billerica, MA, USA). The WACY Software program was used for data baseline subtraction and data acquisition (Edmund Howard, Dave Thomas lab, University of Minnesota, Minneapolis, MN, USA). Reactants were mixed in test tubes in a final volume of 20 *μ*l, and then transferred to capillary (0.6/0.84 mm ID/OD, Vitrocom, Mountain Lakes, NJ, USA) assemblies for EPR measurement. The concentrations shown in the figure legends are final concentrations. All experiments were performed at room temperature and under ambient air except those as specifically indicated. Spectra were acquired with the following parameters: 2 mW microwave power, 1 G peak-to-peak modulation amplitude, 120 G sweep width and 40.96 ms time constant and conversion time.

### TUNEL assay

KYSE510 cells (2 × 10^4^) were seeded into four-chamber polystyrene vessel tissue culture glass slides (BD Biosciences) in 500 *μ*l 10% FBS/RPMI-1640 medium and incubated at 37 °C in a 5% CO_2_ humidified incubator. After culturing for 24 h, different concentrations of HOI-02 were added to each chamber. After incubation for another 24 h, cells were fixed in 4% paraformaldehyde in PBS for 25 min at room temperature (25 °C). Cells were washed three times in PBS for 10 min each time and then stained using the DeadEnd Fluorometric TUNEL System (Promega) according to the manufacturer's protocol. All of the samples were counterstained with 4',6-diamidino-2-phenylindole (DAPI). The number of TUNEL-positive cells was determined by laser scanning confocal microscopy (Nikon C1^S1^ Confocal Spectral Imaging System, Nikon Instruments Co., Melville, NY, USA) using a CFI Plan Fluor × 20 objective and then analyzed using the EZ-C1 (v3.20) software program (Nikon).

### Anchorage-independent growth assay

KYSE30, KYSE450 and KYSE510 cells (8 × 10^3^ per well) were suspended in 1 ml Basal medium Eagle (BME), 10% FBS, and 0.33% agar and plated with various concentrations of HOI-02 or HOI-11 on 3 ml of solidified BME containing 10% FBS and 0.5% agar with different concentrations of HOI-02 or HOI-11. Cells were incubated for 12 days and then colony number was determined by microscope and the Image-Pro Plus software (v. 6.1) program (Media Cybernetics, Inc., Rockville, MD, USA).

### Reporter gene activity

KYSE510 cells (5 × 10^4^ per well) were seeded into 12-well dishes and incubated for 24 h. Cells were transfected with the *AP-1 reporter* plasmid (800 ng) and incubated for 36 h and then treated with HOI-02, NAC or GSH for 24 h. Firefly luciferase activities were measured using substrates provided in the reporter assay system (Promega). Transfection efficiency was normalized with a *Renilla* plasmid as an internal control.

### *In vivo* PDX model

Esophageal cancer tissue was collected from a 64-year-old male patient diagnosed with moderate esophageal cancer stage TNM T2N0M0 IIa. This study was approved by the Ethics Committee of Zhengzhou University and the patient whose tumor sample was used in the study was completely informed and gave full consent. PDX models were initiated by subcutaneous implantation of this patient's esophageal cancer fragments (~2–3 mm) coated in Matrigel and implanted through subcutaneous flap incisions. All treatment experiments were performed in C.B-17 severe combined immunodeficient mice, 4 to 6 weeks old at time of PDX injection/implantation. Once tumor volumes reached approximately 150 mm^3^, mice were randomized to treatment with intraperitoneal (i.p.) injections of vehicle or HOI-02. HOI-02 was freshly prepared before each treatment and protected from light before injection. The first group (10 mice) received 100 *μ*l of vehicle only (2% DMSO and 5% Tween-20 in PBS) i.p. every other day for 6 consecutive weeks. The other two groups (10 mice per group) were given 100 *μ*l of HOI-02 (dissolved in 2% DMSO and 5% Tween-20 in PBS) i.p. every other day at a dose of 10 or 50 mg/kg body weight for 6 consecutive weeks. Tumor volume (length × width × depth × 0.52) was measured and body weights were recorded every week. At the end of the study, xenograft tumors were weighed and frozen in liquid nitrogen or fixed in 10% formalin and embedded in paraffin for histological studies.

### Immunohistochemistry analysis

Tumor tissues were embedded in paraffin and subjected to immunohistochemistry. Tissues were deparaffinized and hydrated and then permeabilized with 0.5% Triton X-100/1 × PBS for 10 min. Tissues were hybridized with primary antibodies to detect Ki-67 (1 : 100), phosphorylated c-Jun (1:50), cleaved caspase 3 (1 : 100) or p21 and then with biotinylated goat anti-rabbit IgG or biotinylated goat anti-mouse IgG for p21 (1 : 25) as the secondary antibodies. An ABC kit (Vector Laboratories, Inc., Burlingame, CA, USA) was used to detect protein targets according to the manufacturer's instructions. After developing with 3,30-diaminobenzidine, the sections were counterstained with hematoxylin and analyzed by microscope (200 ×) and the Image-Pro Plus software (v. 6.1) program (Media Cybernetics, Inc.).

### DCFH-DA staining

To determine the level of ROS, histologic sections of esophageal cancers were stained with DCFH-DA (10 *μ*M) in PBS for 30 min. Samples were then rinsed, mounted and analyzed using a fluorescent microscope system and the Image-Pro Plus software (v. 6.1) program (Media Cybernetics, Inc.).

### Statistical analysis

All quantitative data are presented as mean values±S.D. as indicated. Statistical analysis of data was performed using the Student's *t-*test. Differences with a *P*<0.05 were considered significant.

## Abbreviations

AP-1: activator protein-1
ATM: ataxia telangiectasia mutated
BME: Basal medium Eagle
DAPI: 4,6-diamidino-2-phenylindole
DCFH-DA: 2,7-dichlorodihydrofluorescein diacetate
DMPO: 5,5-dimethyl-1-pyrroline-*N*-oxide
DTPA: diethylenetriamine pentaacetate
EPR: electron paramagnetic resonance
FMN: riboflavin-5′-monophosphate
GSH: glutathione
H_2_O_2_: hydrogen peroxide
JNKs: c-Jun N-terminal kinases
NAC: *N*-acetyl cysteine
NADPH: nicotinamide adenine dinucleotide phosphate
PARP: poly ADP-ribose polymerase
PBS: phosphate-buffered saline
PDX: patient-derived xenograft
ROS: reactive oxygen species
SDS: sodium dodecyl sulfate
SOD: superoxide dismutase
TUNEL: terminal deoxynucleotidyl transferase dUTP nick-end labeling
