# Supplementary figures and images for: HOI-02 induces apoptosis and G2-M arrest in esophageal cancer mediated by ROS
Source: Cell Death Dis. 2015 Oct 15;6(10):e1912–. doi: 10.1038/cddis.2015.227 (PMC4632281; doi:10.1038/cddis.2015.227)

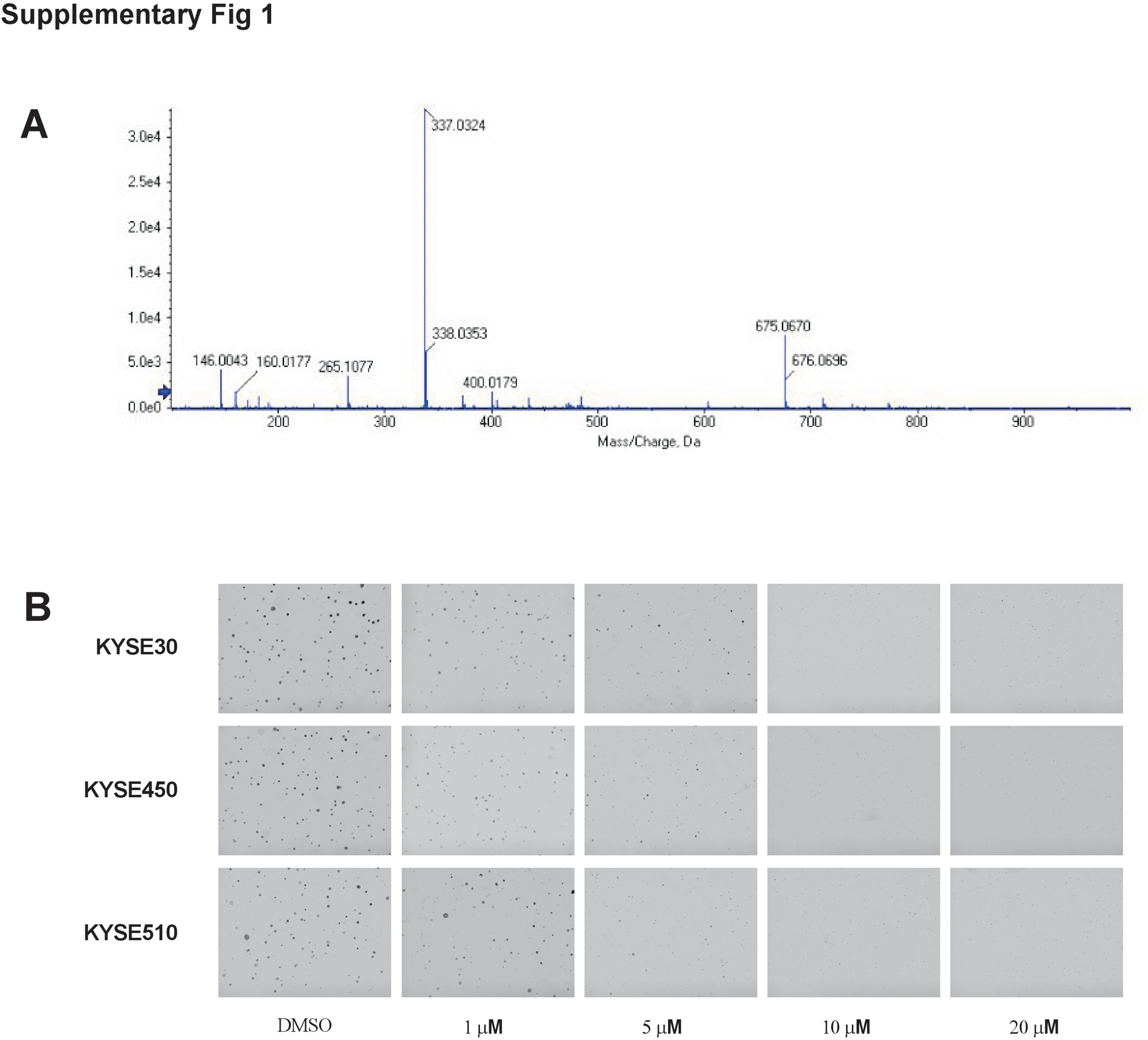

Supplement: Supplementary Figure 1 [file cddis2015227x1.tif]

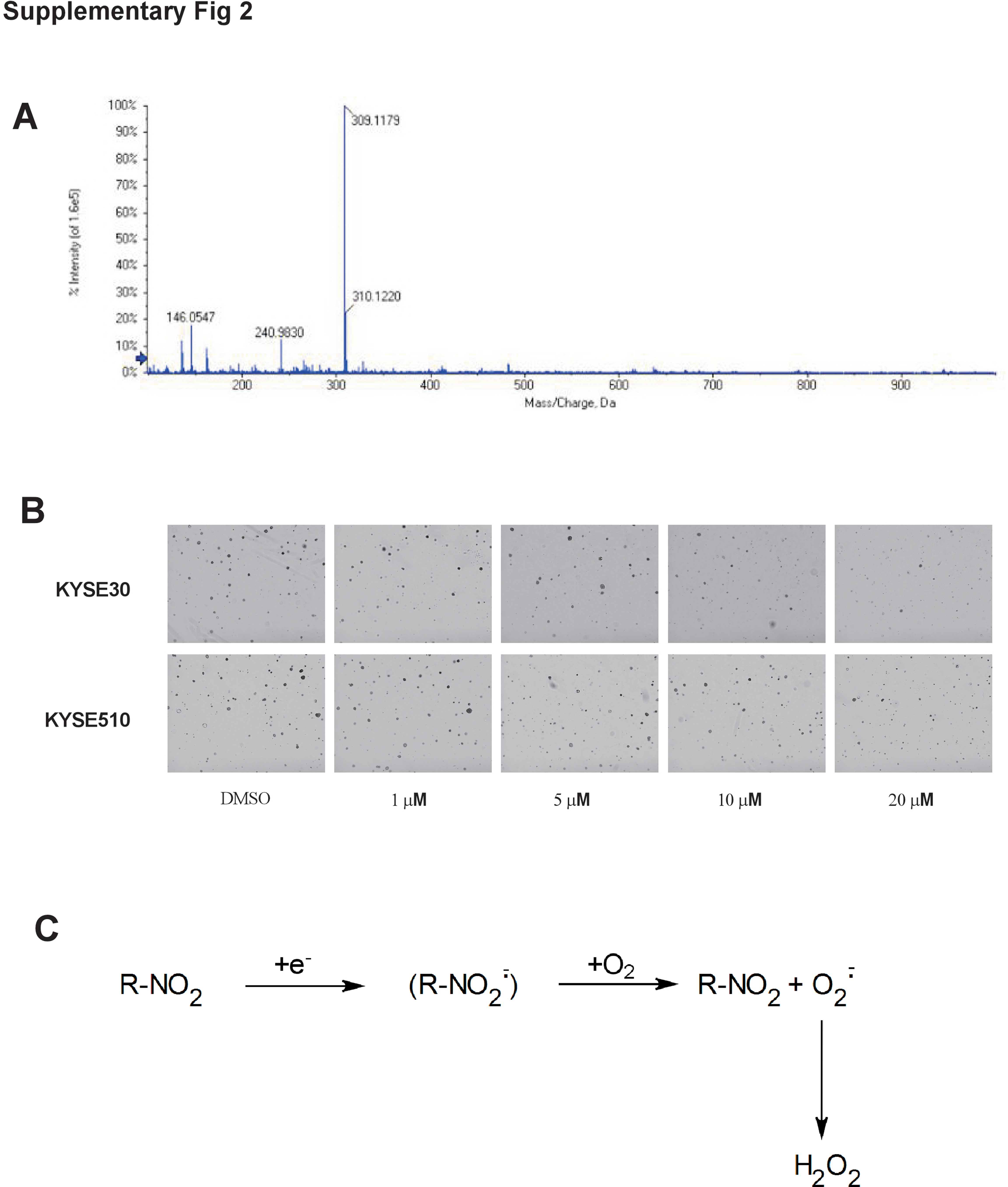

Supplement: Supplementary Figure 2 [file cddis2015227x2.tif]

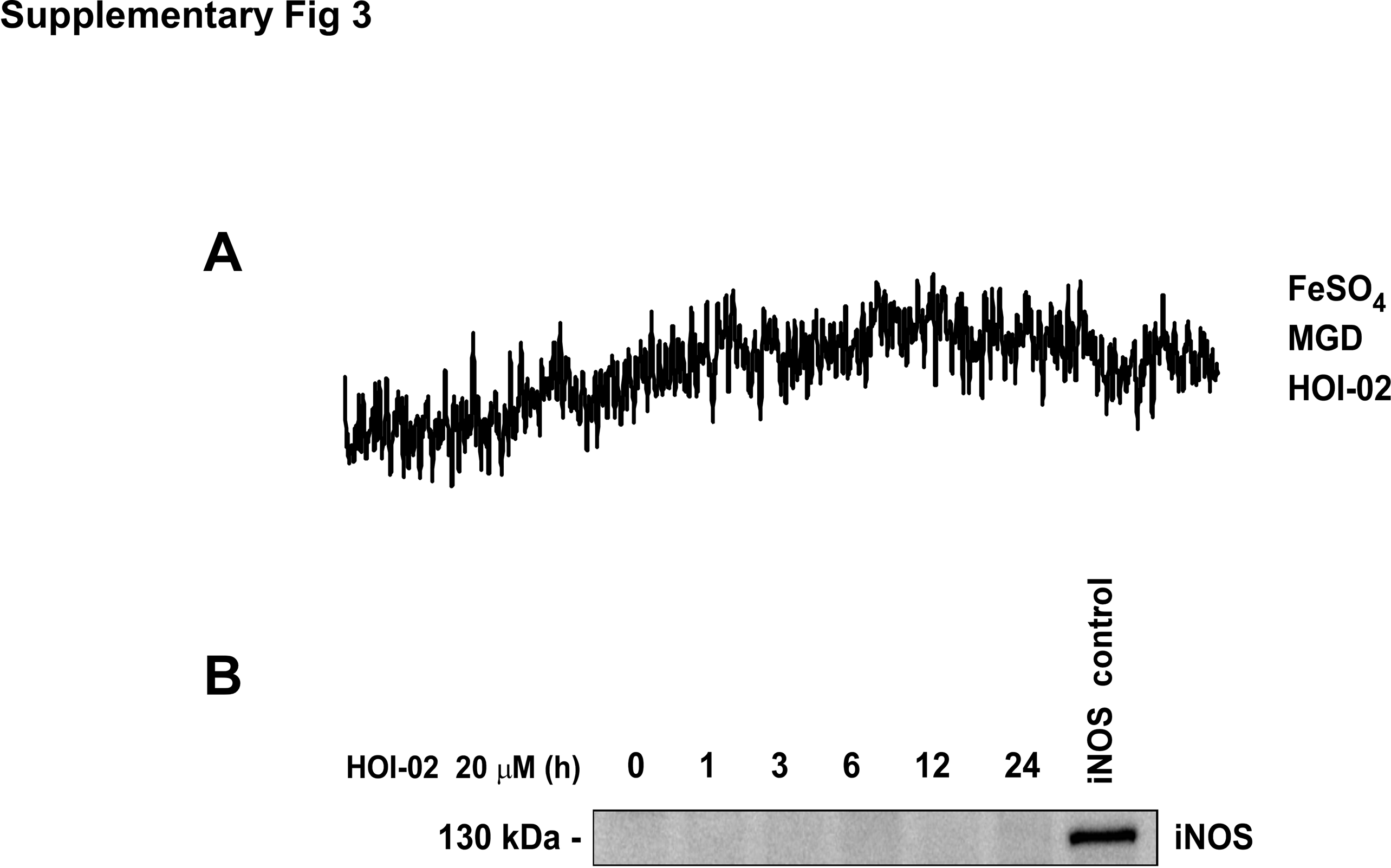

Supplement: Supplementary Figure 3 [file cddis2015227x3.tif]
