# Supplementary material for: HOI-02 induces apoptosis and G2-M arrest in esophageal cancer mediated by ROS
Source: Cell Death Dis. 2015 Oct 15;6(10):e1912–. doi: 10.1038/cddis.2015.227 (PMC4632281; doi:10.1038/cddis.2015.227)
Supplement: Supplementary Figure Legends [file cddis2015227x4.docx]

**Supplemental Figure Legends**

**Supplementary Figure 1.** The MS/MS result of HOI-02 analysis and the effect of HOI-02 on anchorage-independent growth of esophageal cancer cells. (A) The ABSciex TripleTOF^TM^5600 with DuoSpary^TM^ source was used to measure the molecular mass of HOI-02. HOI-02 was dissolved in 50% methanol to a final concentration of 5 μM, and then was directly infused into the mass spectrometer through the ion source. The peak for HOI-02 was detected on negative mode. (B) HOI-02 inhibits anchorage-independent growth of KYSE30, KYSE450 and KYSE510 esophageal cancer cells. Cells were cultured with different concentrations of HOI-02 for 12 days and then colonies were counted.

**Supplementary Figure 2.** The MS/MS result of HOI-11 analysis and the effect of HOI-11 on anchorage-independent growth of esophageal cancer cells. (A) The ABSciex TripleTOF^TM^5600 with DuoSpary^TM^ source was used to measure the molecular mass of HOI-11. HOI-11 was dissolved in 50% methanol to a final concentration of 5 μM, and then was directly infused into the mass spectrometer through the ion source. The peak for HOI-11 was detected on negative mode. (B) HOI-11 was unable to inhibit anchorage-independent growth of KYSE30 and KYSE510 esophageal cancer cells. Cells were cultured with different concentrations of HOI-11 for 12 days and then colonies were counted. (C) The proposed mechanisms of ROS production involves the initial one electron reduction of the nitro group to form the nitro anion radical.

**Supplementary Figure 3.** (A) EPR signals generated in the HOI-02/FeSO_4_-MGD system. EPR spectra were obtained from incubation mixtures containing phosphate buffer (pH 7.4, 50 μM diethylenetriamine pentaacetate [DTPA]) and 20 μM HOI-02, containing 50 μl of 1.9 mM iron (II) sulfate heptahydrate (FeSO4.7H_2_O, prepared fresh by dissolving 0.8 mg in 1 ml DPBS with CaCl_2_ and MgCl_2_) and 50 μl of ammonium N-methyl-D-glucamine dithiocarbamate (MGD, prepared fresh by dissolving 2.7 mg in 500 μl DPBS with CaCl_2_ and MgCl_2_) using a ratio of 1:7. (B) KYSE510 cells (1×10^6^) were treated with 20 μM HOI-02 for 0, 1, 3, 6, 12 or 24 h. The iNOS positive loading control was provided by Cell Signaling Technology, Inc.(Danvers, MA). The expression of iNOS generation was determined by Western blot analysis using a speciﬁc antibody.
